# Past and future of prophylactic ablation of the cervical squamocolumnar junction

**DOI:** 10.3332/ecancer.2015.527

**Published:** 2015-04-29

**Authors:** Silvia Franceschi

**Affiliations:** International Agency for Research on Cancer, 150 Cours Albert Thomas, 69372 Lyon cedex 08, France

**Keywords:** cervical cancer, HPV, screening, prophylactic cervical ablation, ectopy, squamocolumnar junction, squamocolumnar junction cells

## Abstract

HPV vaccination has the potential to prevent the vast majority of cervical cancer cases but cervical cancer screening remains the only prevention strategy in adult unvaccinated women. The range of primary screening tests has expanded to include, in addition to cytology, visual inspection with acetic acid (VIA) and HPV-testing. HPV-testing has the best sensitivity and negative predictive value, and a low-cost HPV test may make large-scale high-quality cervical cancer screening possible in low- and medium-income countries (LMICs). However, on account of its low specificity, HPV-testing would impose the additional burden of triaging HPV+ women using cytology, colposcopy, VIA, or other not yet affordable tests. If minimally invasive treatments that have proven efficacious in HPV+ women with cervical intra-epithelial (CIN) grade 2 and 3 lesions also reduced future cervical cancer risk in lesion-free HPV+ women, the treatment of all HPV+ women would become attractive in LMICs in which screening should be performed as infrequently as possible.

In the pre-mass screening era, gynaecologists widely practiced prophylactic ablation of the columnar epithelium visible on the ectocervix (ectopy) and squamocolumnar junction (SCJ) in the hope of preventing cervical cancer. Favourable outcomes were reported, especially from Finland, but conclusive results were not reached on account of weaknesses in the study methods. Indirect support for prophylactic ablation is provided by the hypothesis that some cells of embryonic origin derived from the SCJ are the source of high-grade CIN and cervical carcinoma, and that they do not regenerate after SCJ ablation. SCJ cell elimination may avoid neoplastic transformation though not HPV reinfection. Randomised controlled trials are gold-standard, but if not feasible, an evaluation of the impact of prophylactic ablation could be done in the framework of large HPV-based screening programmes. Careful follow-up of lesion-free HPV+ women would provide much needed information on the risk-to-benefit ratio of prophylactic ablation.

## Background

Cervical cancer was the first malignancy to become the object of screening programmes. As early as the 1950s, Papanicolaou [1], demonstrated that cytology could be used to identify precancerous lesions thus shifting the emphasis from the identification of women with invasive cervical carcinomas to the identification and treatment of women with cancer precursors. In the 1960s, cervical cytology was widely adopted in many high-income countries and gradually reached a larger number of world populations than any other type of cancer screening [2]. Of note, in countries with high quality programmes of cervical screening and consequently the possibility of treating safely and effectively precancerous lesions, decreases in the incidence and the mortality of cervical cancer were seen [2]. In this way, cervical screening was spared the degree of controversy on ‘overdiagnosis’ that has plagued, for instance, breast cancer screening.

Over the last decade there has been an increase in the variety of tests available for cervical screening, e.g. human papillomavirus (HPV) testing and visual inspection with acetic acid (VIA), thus overcoming some of the limitations of cytology and addressing more closely the needs of the different regions of the world. A global expansion of HPV vaccination may curb the risk of cervical cancer by more than 70% in the cohort of women who are now being vaccinated in their early adolescence. However, no therapeutic vaccines or anti-HPV medical treatments are available and early detection and treatment of precancerous lesions is the only way to avoid cervical cancer in HPV+ women. Between the 1920s and the 1970s, gynaecologists widely performed prophylactic ablation of the squamocolumnar junction (SCJ) and columnar epithelium visible on the ectocervix in an attempt to diminish a woman’s susceptibility to infection and cervical cancer [3, 4].

In the present report, I will briefly outline a few large observational studies of prophylactic ablation and discuss possible synergies between the introduction of HPV-based primary cervical screening, notably in low- and middle-income countries (LMICs), and the possibility of assessing the value of prophylactic ablation.

## Past experience with prophylactic ablation

A large amount of studies have demonstrated the efficacy and safety of cryotherapy [5] and excision [6] of the SCJ and transformation zone in the treatment of cervical intraepithelial neoplasia grade 2 or 3 (CIN2/3). However, in the pre- mass screening era, gynaecologists had been concerned by the vulnerability to infections and cancer of the columnar epithelium visible on the ectocervix (a condition referred to as ectopy and very frequent in young women [7]) and had tried to eliminate it [8]. The importance of cauterisation to a healthy cervix had been advocated since the 1920s [9]. The aim was to shorten the physiological time of replacement of columnar epithelium by squamous epithelium (metaplasia) from many years to a few weeks. A total of 13,897 women attending a private gynaecological practice in the United States were retrospectively assessed with regard to the benefits of prophylactic ablation. All patients possessing a cervix and having had two or more visits were eligible. For ablation, electrodiathermy coagulation had been conducted since 1937 as an office procedure [3]. Pap smear has been used since 1949 in women of age 35 years or older and since 1965 in women of any age. Prophylactic ablation was performed in 6364 patients. Approximately 20% of nulliparous and 80% of parous women had visible ectopy after cleansing with metacresol. Among untreated women, 81 later developed *in situ* or invasive carcinoma (27 per 1000 women). In contrast, three *in situ* and no carcinoma were detected in treated women (0.5 per 1000 women). Peyton *et al* [3] reported some additional side benefits of prophylactic ablation, namely decrease in vaginal discharges, better tolerance of oral contraceptives, and surprisingly, a favourable effect on reproductive outcome. In a multiple-author correspondence that appeared as comments on the paper, other gynaecologists raised some doubts about the good comparability of treated and untreated women and reported in their experience, cases of cervical stenosis. Some deplored the lack of systematic histopathological and cytological assessment of cervical lesions at the time, but overall the usefulness of prophylactic ablation was widely supported.

A little later, prophylactic ablation was also widely used in Italy, especially by a team in Milan [10, 11]. Mario Sideri was an active member of the team both as a student and as a junior doctor, especially in studies on ectopy in very young women [12]. The team followed up 4358 cervical cancer-free and predominantly young women who had attended the colposcopy clinic from 1964 to 1971. Ectopy, with or without metaplastic areas, was colposcopically diagnosed when there was no homogeneous uptake of Lugol staining, i.e. iodine-light cervix. A total of 3151 women with iodine-light cervix underwent prophylactic ablation. Post-treatment colposcopy was available for 1786 women and showed return to iodine-dark cervix in the vast majority of treated women (97%). At the end of a 15-year active or passive follow-up, two squamous-cell carcinomas and one adenocarcinoma were reported in the 3151 treated women and three squamous-cell carcinomas in the 1105 untreated women (these included, however, 443 women with iodine-dark cervix at baseline). The authors concluded that cervical cancer was an especially rare event among the well-screened women in their study, the difference in cancer risk by prophylactic ablation was neither significant nor free from selection bias.

The only population-based report on prophylactic ablation was a linkage study involving records from the Finnish Mass Screening Registry and National Cancer Registry [13]. A total of 429,832 women had a first time cytology test in the 1963–1972 period within the framework of the Finnish cervical cancer screening programme and 67,321 of these women (16%) had a history of prophylactic ablation by electrodiathermy coagulation. This percentage was highest (>20%) in women age 25–39 years and 3% in those above age 55. Women not subjected to prophylactic ablation had a six-fold increased prevalence ratio (PR) for invasive cervical cancer and four-fold increased PR for *in situ* carcinoma and severe dysplasia (Table 1). Published data only allowed the computation of crude PR and 95% confidence intervals but an age-stratified table in the report provided reassurance on the consistency of the beneficial effect of prophylactic ablation within age-groups (from 30–34 to 50–54).

In conclusion, past studies of prophylactic ablation illustrate the substantial interest in accelerating the replacement of the columnar epithelium with squamous epithelium on the ectocervix. A few studies showed some favourable results in the pre-mass screening era. They did not reach, however, conclusive results on account of weaknesses in the study methods, i.e., lack of randomisation, insufficient follow-up, and inadequate adjustment for confounding.

## HPV-based primary screening

The advent of HPV testing in primary cervical cancer screening was a welcome improvement. A large number of studies in which women were screened with both cytology and HPV testing, and referred to colposcopy if either test was positive, showed that HPV DNA has higher sensitivity and negative predictive value than cytology for detecting CIN2+ [14]. Three [15–17] out of four European randomised controlled trials [15–18] that compared women who were screened with and without HPV testing showed greater detection of CIN2 and CIN3 with HPV-based testing compared to cytology-based screening at the first round. All four studies with longitudinal data showed fewer CIN2 and CIN3 at the second round of screening (after three to five years) in the group initially screened by HPV, compared to the one screened by cytology. Taken together, these two results suggest that HPV-based screening in women above age 30 or 35 provided earlier detection of cervical lesions that would have persisted at the second screening round (i.e. clinically relevant lesions). The reduced detection of CIN2+ in the second round shows that prolonged screening intervals are safe in HPV-negative women and short intervals should be discouraged to avoid over-referral for recent and often regressive HPV infections [14]. As a proof of principle, the combination of European trials demonstrated, despite the low number of invasive cervical cancers in the trials, a significantly larger protection against invasive cervical cancers through HPV-based testing compared to cytology-based screening [19].

The main drawback of using HPV testing in screening is a nearly two-fold higher number of screening-positive women [20] on account of the inferior specificity of currently available HPV assays in comparison to cytology. The problem of low specificity obviously becomes more severe in populations in which HPV infection is very frequent, e.g. young women, LMIC populations with high HPV prevalence, and HIV-infected women [21]. A range of different approaches to the triage of women who test positive for high risk (hr) HPV types exist including repetition of HPV test, cytology (gold-standard), p16 immunostaining, HPV genotyping. Triage is necessary to reduce the number of colposcopical examinations and cope with limited availability of colposcopical skills in many countries and with the inaccuracy of the technique in some circumstances, e.g. the false negative findings that are associated with thin cervical epithelium [22]. A novel immunochromatographic test that detects E6 oncoproteins of some hrHPV types [23] and experimental molecular tests based on DNA hypermethylation of certain tumourrelevant genes in cervical cancer and precancerous cervical lesions [24, 25] offer hopes of full molecular screening programmes applicable to self-collected cell samples.

For the moment, however, none of the recommended or promising triage methods is suitable or available in low-income countries on account of the cost and complication of triage tests. This problem threatens one of the greatest advantages of HPV testing over cytology i.e., its potential applicability in LMICs. At its best, cytology can be nearly as good as HPV testing [18], but maintaining such high-quality cytology has been shown to be very difficult even in high-income countries [26]. A few large trials of HPV test-based screening in Africa and Asia have shown consistently that HPV DNA testing followed by cryotherapy reduced the incidence of CIN2+ [5] and mortality from cervical cancer [27], and was superior to cytology [27] and VIA [5, 27].

VIA, with or without visual inspection with Lugol’s iodine (VILI), is currently endorsed as a method to triage hrHPV+ in low-income countries in national protocols [28] and by WHO guidelines [29]. VIA has often been described as a reasonably sensitive (79%) test for CIN2+ that lacks specificity (85%) [30]. However, the performance of VIA in primary screening can vary enormously and quality control is difficult. The bias-corrected sensitivity of VIA for CIN2+ was <45% in some studies [31–33] and decreased as women grew older [23]. The advantages of HPV-testing in terms of sensitivity and negative predictive value may therefore be lost if a relatively low-sensitivity method such as VIA is used to select the women who need treatment. Eventually, the trade-off of advantages and disadvantages in offering some treatment to the majority if not all hrHPV+ women depend on whether such treatment may diminish the risk of developing CIN2+ and especially CIN3, among hrHPV+ women who are VIA-negative.

## New rationale for prophylactic ablation

A new population of cells of embryonic origin that derive from the SCJ of the cervix (henceforth referred to as SCJ cells) have recently been characterised [34, 35]. These cells have a unique cuboidal morphology and express specific biomarkers, i.e. Krt7, AGR2, CD63, MMP7, and GDA [34]. Unlike the stratified epithelia of the cervix and anogenital tract, SCJ cells are not permissive for the entire HPV life cycle but may harbour the virus for extended periods of time [36]. Most importantly, Herfs *et al* [34, 35] have proposed that SCJ cells are the source of most, if not all, CIN2/3 and cervical carcinomas, and that they do not regenerate after ablation of the SCJ and the transformation zone.

In an effort to confirm their earlier findings, Herfs *et al* [37] assessed the outcome of the loop electrosurgical excision procedure (LEEP) in 131 women who had CIN. Infection with hrHPV at enrolment (n = 125) and CIN2/3 in the cone biopsy (n = 128) had been detected in the vast majority of study women. During follow-up (median: 89.5 weeks) CIN was detected in 16 women (12.2%) and two groups were distinguished: 1) Residual disease (four women): CIN2/3 that were found at the first follow-up visit after LEEP, and closely resembled the initial CIN in grade, and type of HPV infection suggesting incomplete excision; and 2) Delayed recurrences (12 women): lesions that manifested at later visits, were all CIN1, and frequently positive for different HPV types than those detected at enrolment. Interestingly, all residual CIN2/3 but one (also negative at baseline) were still positive for SCJ cell markers after LEEP versus none from recurrent CIN1 group. The findings from Herfs *et al* [37] suggest, therefore, that the elimination of SCJ cells does not prevent HPV infection and its morphological manifestation (ClN1) but may avoid neoplastic transformation.

## Knowledge gaps

Alternative and less invasive ways than prophylactic ablation may exist to prevent or reverse the HPV-driven malignant transformation process in the cervix, e.g. a therapeutic HPV vaccination (not dealt with in the present article) or hormone modulation. An important knowledge gap in the natural history of SCJ cells and by and large the HPV-driven cervical carcinogenicity is the role of female hormones [38]. The cervix is very sensitive to changes in levels of endogenous and exogenous female hormones during a woman’s life [7]. The rise in ovarian activity after menarche stimulates metaplasia in the adolescent cervix through pH lowering and direct stimulation of subcolumnar reserve cells and their maturation into squamous epithelium [7]. However, a longitudinal study of incident HPV infection among young women [39] found a reduced infection rate among those for whom first sexual intercourse was close to menarche, suggesting that the size and vulnerability of the ectopy increases rather than diminishing in the first years after menarche. A follow-up study on 13–21 year old women showed that rapid maturation of the cervix rather than the size of ectopy increased the risk of HPV16 infection [8]. In respect to cervical cancer, multiparous women [40] and long-term users of oral contraceptives [41] have an increased cervical cancer risk after adjustment for sexual habits. In addition, incidence rates of the disease in unscreened populations have been shown to stop rising after approximately 45 years of age i.e. when peri-menopausal hormonal changes start [42]. Both full-term pregnancies and oral contraceptives involve exposure to high levels of oestrogens and progesterone, and which of the two groups of hormones is associated with cervical cancer is unclear [43]. However, in hybrid mice [44] and HPV transgenic mice [45, 46], high oestrogen levels have been shown to increase cervical cancer. In HPV transgenic mice, cervical cancer can be controlled by treatment with raloxifene, an oestrogen-receptor antagonist [47]. Data on the incidence of *in situ* and invasive cervical cancer in the participants of two randomised clinical trials on the effect of raloxifene and tamoxifen (an oestrogen-receptor agonist in the genital tract) were inconclusive [48] on account of the very low incidence of cervical cancer in the very well screened middle-age women who participated in the trials.

## Joint evaluation of HPV-based screening and prophylactic ablation

If the findings by Herfs *et al* [37] that ablation of SCJ cells reduces the risk of subsequent CIN2+ in hrHPV+ women are confirmed by other groups and larger studies, there would be a strong incentive to re-evaluate prophylactic ablation, especially in LMICs in which the success of cervical cancer screening depends on the possibility of performing it well, but as infrequently as possible (ideally once or twice in a lifetime). In fact, the report by Herfs *et al* [37] ends with a plea to start randomised controlled trials to determine the safest ways to perform prophylactic ablation and establish its efficacy in the more cervical cancer-prone women (e.g. HIV-infected/multiple-sexual partner women).

As in the past, a large randomised trial of prophylactic ablation would be very challenging especially because of concerns about adverse pregnancy outcomes in women who still wish to have children. A meta-analysis [49] and large prospective study [50] of women treated for CIN showed an association of pre-term delivery with cold knife or laser conisation but provided reassuring findings for loop excision and cryotherapy. Reassuringly, no increase in HIV shedding was found after cryotherapy in HIV-infected women who were taking antiretroviral therapy [51].

A possible alternative to randomised trials may be a careful evaluation of the impact of prophylactic ablation in the framework of large screening programmes [4]. The new ‘World Health Organisaton (WHO) guidelines for screening and treatment of precancerous lesions for cervical cancer prevention’ in LMICs [29] endorse HPV-based screening. Treatment with cryotherapy is recommended in either all HPV+ women or only those who are VIA+. A randomised screening trial conducted in 6555 women 35 years or older in South Africa [5] had provided some support to the ‘treat-all’ alternative. The HPV-based screen-and-treat approach significantly reduced CIN2+ lesions through 36 months in both HIV-negative (relative risk = 0.31; 95% confidence interval, CI: 0.20–0.50) and HIV+ women (relative risk = 0.20; 95% CI: 0.06–0.69) [52]. The risk-to-benefit ratio of the approach was highly favourable: there was only one serious complication (bleeding leading to transfusion in an HIV+ woman). Nearly all participants stated that they would recommend this type of screening programme to friends and family. Cytology was not evaluated in the trial and it is therefore highly probable that a substantial fraction of HPV+ women who underwent treatment had no cervical abnormalities at enrolment and therefore, received what we are referring to as prophylactic ablation. Of interest, Kuhn *et al* [52] showed that CIN1 incidence after cryotherapy was not reduced in HIV+ women. This observation is consistent with the hypothesis proposed by Herfs *et al*. [37] and the findings by Hwang *et al* [8] that the elimination of SCJ cells and ectopy in high-risk women may protect from neoplastic transformation rather than from new HPV infection or re-emergence of latent infection.

## Conclusions

In order to advance current knowledge on prophylactic ablation, a study could be envisaged in which cryotherapy is performed in a sufficiently large subset of hrHPV+ women showing columnar epithelium on the ectocervix with or without metaplasia but no evidence of cervical precancerous or cancerous lesions at VIA/VILI or colposcopic examination. The ideal age target for prophylactic ablation needs to be defined but it would likely be from age 30 or 35 to 40 or 45. Women who have evidence of cervical precancerous or cancerous lesions would be managed according to well-established protocols [29]. In women who undergo prophylactic or therapeutic ablation, a biopsy should be taken immediately prior to cryotherapy for *post-hoc* diagnostic confirmation. Prophylactic ablation would not be performed in women with mature metaplasia or in women in whom the SCJ were not visible. Of note, the distinction of mature from immature cervix is difficult using VIA/VILI pointing to the additional usefulness of colposcopical examination and the desirability of colposcopical skill improvements in LMICs [53]. Follow-up of lesion-free women and comparison of the incidence of precancerous or cancerous lesions across study groups would help us learn more about the role of prophylactic ablation and possibly SCJ cells. The study would also provide much needed information on the risk-to-benefit ratio of treating all HPV+ women in settings in which laborious and expensive methods of triage of an HPV+ test are difficult to put in place.

## List of abbreviations used

CIN: cervical intraepithelial neoplasia
HPV: human papillomavirus
hr: high-risk
LEEP: loop electrosurgical excision procedure
LMICs: low- and middle-income countries
PR: prevalence ratio
SCJ: squamocolumnar junction
VIA: visual inspection with acetic acid
VILI: visual inspection with Lugol’s iodine

## Conflict of interest

No potential conflict of interest is disclosed by the author.

## Figures and Tables

**Table 1.** Prevalence ratio of cervical neoplasia by history of electrodiathermy coagulation, Finland, 1963–1972, Kauraniemi *et al* 1978 [13].

Malignancy	Electrodiathermy coagulation Yes N (%)	Electrodiathermy coagulation No N (%)	Prevalence ratio^1^	95% Confidence intervaI^1^
Normal	67 222 (99.9)	360 417 (99.4)	1.0^2^	–
Dysplasia levis	33 (0.05)	451 (0.12)	2.5	1.8–3.6
Severe Dysplasia	25 (0.04)	554 (0.15)	4.1	2.8–6.2
Carcinoma *in situ*	34 (0.05)	791 (0.22)	4.3	3.1–6.1
Microinvasive	0 (0.00)	60 (0.02)	∞	2.9–∞
Invasive carcinoma	7 (0.01)	238 (0.07)	6.3	3.0–13.4
Total	67 321	362 511		

1 Calculated from Table IV in Kauraniemi *et al* 1978.

2 Reference category
